# Pearls and Pitfalls of Isolating Rat OPCs for In Vitro Culture with Different Methods

**DOI:** 10.1007/s10571-023-01380-2

**Published:** 2023-07-05

**Authors:** Justyna Janowska, Justyna Gargas, Joanna Sypecka

**Affiliations:** NeuroRepair Department, Mossakowski Medical Research Institute Polish Academy of Sciences, Pawinskiego 5, 02-106 Warsaw, Poland

**Keywords:** Oligodendrocytes, Oligodendrocyte progenitor cells, Cell isolation, Primary cell culture, Mixed glial cultures, A2B5

## Abstract

There are several in vitro models to study the biology of oligodendrocyte progenitor cells (OPCs). The use of models based on induced pluripotent stem cells or oligodendrocyte-like cell lines has many advantages but raises significant questions, such as inaccurate reproduction of neural tissue or genetic instability. Moreover, in a specific case of studying the biology of neonatal OPCs, it is particularly difficult to find good representative model, due to the unique metabolism and features of these cells, as well as neonatal brain tissue. The following study evaluates two methods of isolating OPCs from rat pups as a model for in vitro studies. The first protocol is a modification of the classical mixed glial culture with series of shakings applied to isolate the fraction of OPCs. The second protocol is based on direct cell sorting and uses magnetic microbeads that target the surface antigen of the oligodendrocyte progenitor cell—A2B5. We compared the performance of these methods and analyzed the purity of obtained cultures as well as oligodendrocyte differentiation. Although the yield of OPCs collected with these two methods is similar, both have their advantages and disadvantages. The OPCs obtained with both methods give rise to mature oligodendrocytes within a few days of culture in ITS-supplemented serum-free medium and a 5% O_2_ atmosphere (mimicking the endogenous oxygen conditions of the nervous tissue).

**Graphical Abstract:**

*Methods for isolating rat OPCs* In the following study we compared methods for isolating neonatal rat oligodendrocyte progenitor cells, for the studies on the in vitro model of neonatal brain injuries. We evaluated the purity of obtained cell cultures and the ability to maturate in physiological normoxia and serum-free culture medium.

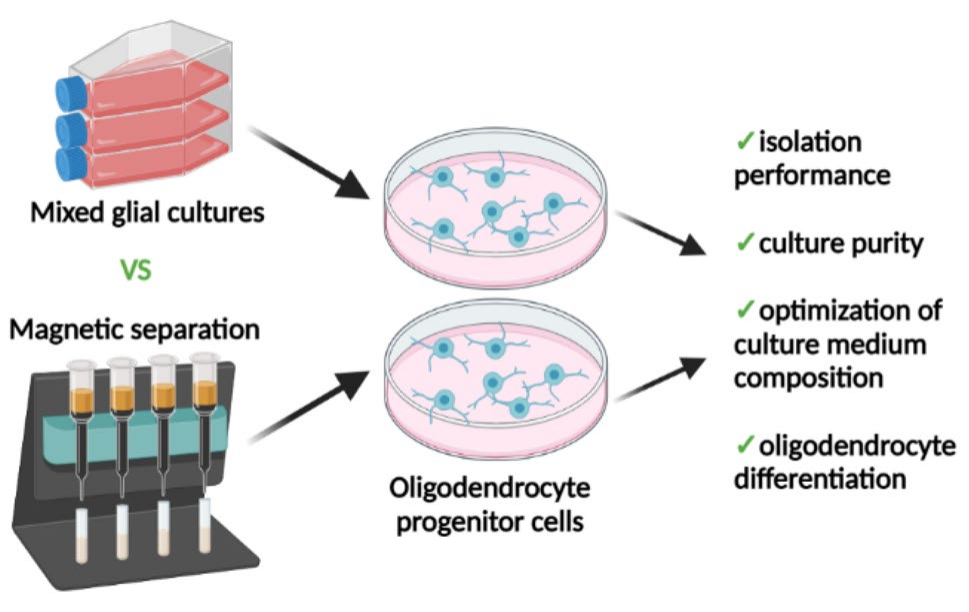

**Supplementary Information:**

The online version contains supplementary material available at 10.1007/s10571-023-01380-2.

## Introduction

Oligodendrocyte progenitor cells (OPCs) have received increasing attention in recent years in the context of testing therapies for various neurodevelopmental and neurodegenerative diseases, as well as injuries to the central nervous system. The main role of these cells is to go through the tightly regulated and multistage process of maturation and eventually produce myelin sheaths around the axons of neurons (Kuhn et al. 2019). This process is most intense during the development of the central nervous system. After this period, OPCs that reside within the white and gray matter throughout adulthood, retain their ability to myelinate, but can also start proliferation and support neural tissue under various pathophysiological conditions (Fernandez-Castaneda and Gaultier 2016).

When looking for treatments of leukodystrophic or demyelinating disorders, as well as to study the biology of oligodendrocyte lineage cells, in vitro models are among the most useful tools. Recent advances in the neuroscience offer the possibility to reduce the number of animals in basic research by conducting in vitro experiments on brain organoids (Chesnut et al. 2021), neurons and glial cells generated from human induced pluripotent stem cells (Chamling et al. 2021; Tukker et al. 2016, 2020; Wang et al. 2013; You et al. 2022) or mesenchymal stem cells derived from the umbilical cord (Sypecka et al. 2017; Sypecka and Sarnowska 2015). Oligodendrocyte lineage cells can be also differentiated directly from neural stem cells derived from brains of rodents of different age (Baldassarro et al. 2020; Li et al. 2019; Samper Agrelo et al. 2020), human aborted fetuses (Lu et al. 2015; Wang et al. 2018; Ye et al. 2022) as well as from adults (Marei et al. 2018) Another useful models are immortalized cell lines, for example, growth factor-dependent cell line of rat oligodendrocyte progenitors—CG4 (Tontsch et al. 1994), non-immortalized rat oligodendrocyte precursor line—OL-1 (Lagarde et al. 2007) or immortalized mouse OPCs—Oli-neu (Naffaa et al. 2022).

Depending on the topic of the research, the appropriate model can be chosen. Methods employing human stem cells to generate oligodendrocyte cells are being improved but still require usually dozens of days in culture (García-León et al. 2018; Xu et al. 2022). There are also disadvantages that are difficult to overcome, such as the high cost of production and the potential genomic instability or the impact of the characteristics of an individual donor on the cell physiology (Slanzi et al. 2020). On the other hand, despite the ease of maintaining cell lines in everyday laboratory practise, there are some concerns about inaccurate replication of the tissue of origin (Lorsch et al. 2014). What is moreover important to notice, that recent studies on the proteomics of OPCs reveal, that the majority of important proteins is expressed differently in neonate vs adult—derived cells, both human and rodent (de la Fuente et al. 2020; Fernandes et al. 2021).This means that the results obtained on cell lines or oligodendrocyte cells derived from iPSCs may be inadequate in terms of biology, particularly the metabolism of neonatal tissue. Thus, it is particularly challenging to search for new therapies for neurodevelopmental disorders, for example perinatal hypoxia, which can lead to disorders of white matter formation (Janowska et al. 2020). In order to study the biology of oligodendrocytes in developing brain it is important to use neonatal-derived tissue. Primary OPCs isolated directly from rat brains, cultured in optimized medium and physiological oxygen conditions, maintain the most important functions and characteristics observed in vivo.

The aim of the study was to establish an in vitro model for conducting experiments on neonatal OPCs for further studies on the impact of brain injuries in the perinatal period that affect the development of white matter, particularly white matter injury after neonatal hypoxia–ischemia. We evaluated two methods of neonatal rat OPCs isolation—the classical method developed by MCarthy and de Vellis (1980) including an intermediate step of mixed glial culture with modifications, (hereafter referred to as MGC method) and direct isolation with A2B5 magnetic microbeads (MB method).

The first method requires the preparation of homogenates of whole brains plated onto flasks. It takes advantage of the properties of mixed culture to grow in layers—astrocytes at the bottom of the culture flask, and on top of them, oligodendrocytes/OPCs, and microglia. Due to the different adhesive properties, glial fractions can be separated during sequential shaking of the flasks.

In the MB method, the enzymatically digested brain tissue samples are incubated with magnetic microbeads, conjugated to highly specific antibodies against the A2B5 surface antigen and glial progenitor marker (Baracskay et al. 2007; Baumann and Pham-Dinh 2001; Gard and Pfeiffer 1990). Cell suspension then flows through the column filled with ferromagnetic spheres that amplify the magnetic field. Columns placed in a magnet retain positively labelled cells and let flow through the unlabeled, negative fraction. Removing the column from the magnetic field allows the collecting A2B5–labeled cells.

In the following study, we present optimized methods of OPCs isolation and results of the evaluation of growth and homogeneity of obtained OPCs cultures and screening for the presence of astrocytes, microglia, and neurons.

Replacing the classical MGC method of OPCs isolation with MB method would allow to reduce the use of animal-origin media, as well as would help to overcome the problem of great amount of plastic disposables and culture dishes that is necessary to run 2 weeks of mixed glial cultures. Primary OPCs cultures exposed to oxygen–glucose deprivation conditions could be a good model in preclinical studies on potential therapies for neonatal hypoxia–ischemia, being an alternative to the classical Rice-Vanucci rat in vivo model of hypoxic-ischemic injury (Rice et al. 1981).

## Animals

In order to prepare cultures, brains removed from Wistar rats of both sexes were used. Animals were killed by decapitation, according to Directive 2010/63/EU of the European Parliament and of the Council of 22 September 2010 on the protection of animals used for scientific purposes. Animals were 1–2 days old (MGC method) or 1-day old (MB method). For these experiments, 6 separate isolations were performed by both methods, each using 3–6 rats (*n* = 60).

## Materials and Methods

### Isolation of OPCs from Mixed Glial Cultures (MGC Method)

**Fig. 1:**
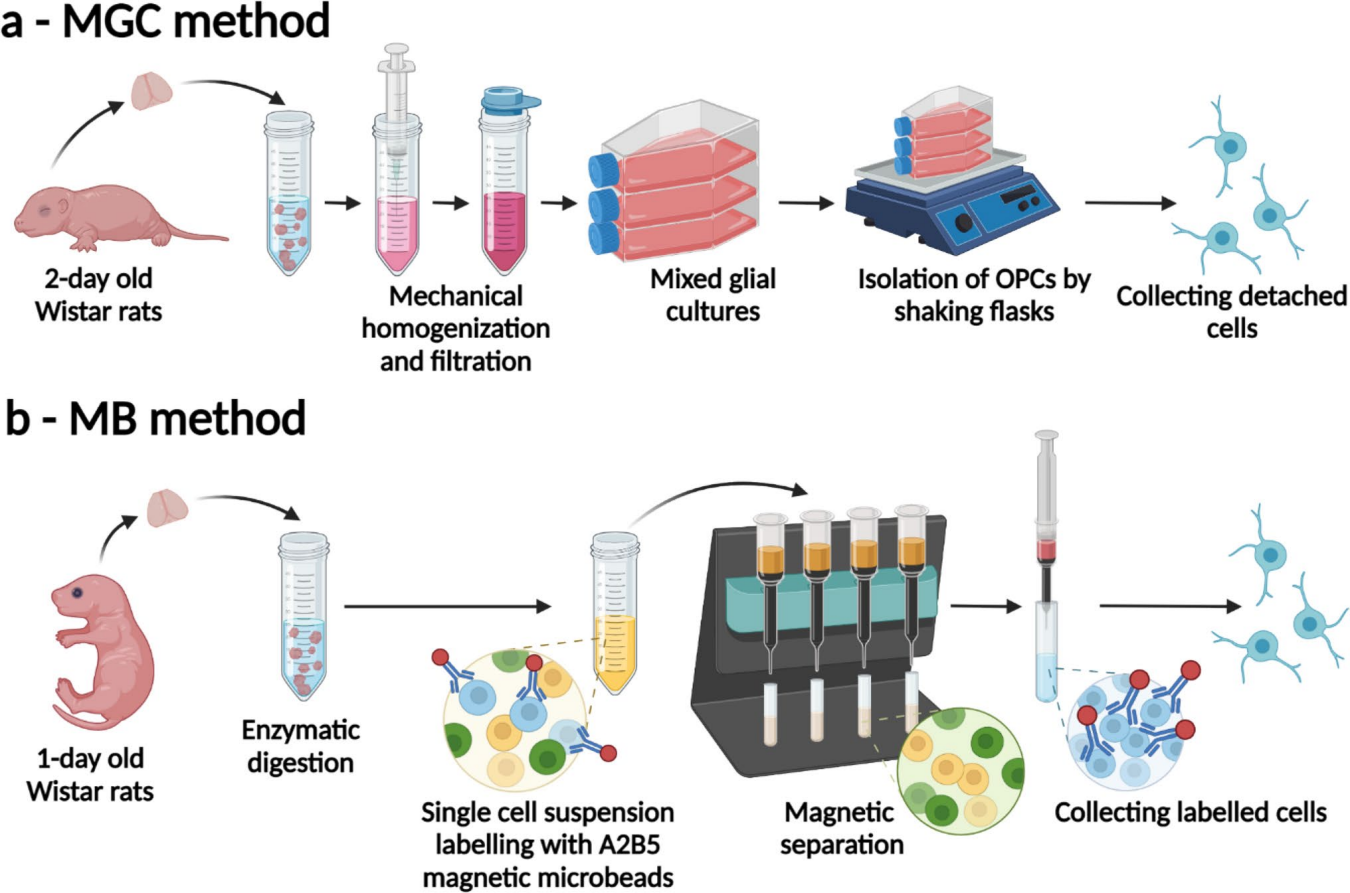
Schematic representations of the OPC isolation methods used in this experiment. **a** Mixed glial culture (MGC) method. **b** Microbeads (MB) method. Figure created with BioRender.com

The method of establishing mixed glial cultures was based on methods described previously (Chen et al. 2007; Sypecka and Sarnowska 2014) with minor modifications. Cerebral hemispheres were mechanically dissociated in a culture medium using a Pasteur pipette, then with an automatic pipette with a 1 ml tip, and finally, using a syringe with a needle. The medium for homogenization and subsequent culture consisted of DMEM Glutamax high glucose (Gibco), 10% fetal bovine serum (FBS; Gibco), and 1% AAS (Sigma-Aldrich). The suspension obtained was filtered through a cell strainer with a mesh size of 40 µm and seeded onto poly-l-lysine coated (0.7 µg/cm^2^; Sigma-Aldrich) culture flasks (Nunc EasY T75, ThermoFisher) at a density of 5 × 10^4^ cells/cm^2^, in a 15 ml volume of the culture medium. The prepared mixed glial cultures were maintained in an incubator under standard conditions (37 °C, 21% O_2_, 5% CO_2_) with medium change every 2–3 days. After 11–13 days of culture, the isolation of OPCs was carried out using a mechanical method that takes advantage of the different adhesive properties of the various glial fractions. For this purpose, culture flasks in an incubator were placed on an orbital shaker operating at 160 rpm. After 1 h, the resulting supernatant with the microglia was removed and the flasks were refilled with fresh medium. Cultures were shaken for an additional 18–20 h. The supernatant obtained contained detached OPCs. The supernatants were pooled and centrifuged for 5 min at 1200 rpm. After removing the filtrate, the remaining pellet was washed with phosphate buffered saline (PBS, Gibco) and centrifuged again. The cells obtained were resuspended in culture medium ‘OLIGO’ [DMEM Glutamax high glucose (Gibco), 1% ITS (Gibco) 1% AAS (Sigma-Aldrich)]. A schematic of the procedure is shown in Fig. 1a. The remaining flasks can be in meantime refilled with fresh culture medium and shaken again for 18–20 h in order to collect the second portion of OPCs. After one short shaking for collecting microglia, followed by shakings for collecting OPCs, the remaining fraction consists mostly the astrocytes. These cells can later be trypsynized and used for other experiments. The characteristics of microglia and astrocytes obtained by this method are described elsewhere (Gargas et al. 2022).

### Isolation of OPCs with Magnetic Microbeads (MB Method)

This method of isolation of OPCs is modified from the method described by the manufacturer of magnetic microbeads, Miltenyi Biotec. It requires using enzymatic digestion instead of mechanical homogenization. The brain hemispheres were devoid of meninges and washed with cold, sterile HBSS (w/o) (Hank’s Balanced Salt Solution without calcium and magnesium; Gibco). The tissue was cut into small pieces and the tissue of three brains was placed in a warm solution of an enzyme mix 1 (100 μl enzyme P + 3.8 ml buffer X) from Neural Tissue Dissociation Kit P (Miltenyi). The suspension was mixed with a Pasteur pipette and incubated for 15 min at 37 °C in a rotator. Meanwhile, the enzyme mix 2 was prepared by mixing 20 μl enzyme A with 40 μl buffer X (components of Neural Tissue Dissociation Kit P; Miltenyi). Enzyme mix 2 was added, the suspension was mixed and incubated for the following 10 min at 37 °C in the rotator. The suspension was pipetted with an automatic pipette with a 1 ml tip and incubated for 10 min at 37 °C in the rotator. The suspension was then filtered through cell strainers with a mesh size of 70 µm and after—40 µm. The tubes and strainers were washed with 10 ml of cold HBSS (w) (Hank’s balanced salt solution with calcium and magnesium; Gibco). The suspension was then centrifuged for 10 min at 300×*g*. The supernatant was discarded and the cell pellet was suspended in 280 μl of sterile blocking solution of PBSA [0.5% Bovine serum albumin (BSA; Sigma) in PBS (Gibco)]. The cells were thoroughly suspended in the solution and incubated for 10 min at 4 °C. After this time, anti-A2B5 magnetic microbeads were added (80 μl), well mixed, and incubated for the following 15 min at 4 °C. Tubes were then filled with 8 ml PBSA, mixed and centrifuged for 10 min at 300×*g*. During centrifugation, two pieces of MACS MS columns (Miltenyi) were placed in MACS magnetic separators (Miltenyi) and washed with 500 μl PBSA. Cells after centrifugation were suspended in 1 ml of PBSA and mixed well. The suspension was applied onto two columns, 500 μl each. The columns were then washed three times with 500 μl PBSA, each time after emptying the column reservoir. During this process the negative fraction can be collected to isolate other cell types, for example immature oligodendrocytes, using anti-O4 microbeads (Miltenyi), or microglia, using anti-CD11b/c microbeads (Miltenyi). After the last wash, columns were removed from a magnetic field, 1 ml of culture medium was applied and using the plunger, cells labelled with A2B5 magnetic microbeads (positive fraction) were removed into an eppendorf tube. Culture medium (“PROLIF”) consisted of: MACS Neuro Medium (Miltenyi), 0.5 mM l-glutamine (Sigma), 1% ITS (Gibco), 1% AAS (Sigma-Aldrich), 10 ng/ml Human PDGFAA (Miltenyi), 10 ng/ml Mouse FGF-1 (Miltenyi). A schematic of the procedure is shown in Fig. 1b.

### OPCs Culture Conditions

The OPCs obtained with both methods were seeded at a density of approximately 3 × 10^4^ cells/cm^2^ into 24-well plates (Nunc, ThermoFisher) with poly-l-lysine-coated glass coverslips for immunofluorescencent staining or 6-well plates (Nunclon) for qPCR and cultured under physiological normoxia (37 °C, 5% O_2_, 5% CO_2_).

In order to evaluate the effect of different compositions of culture medium on OPCs differentiation, cells obtained with the MB method after 2 days in culture were then cultured in different media: ‘PROLIF – PDGFR-AA and FGF’ [MACS Neuro Medium (Miltenyi), l-glutamine 0.5 mM (Sigma), 1% ITS (Gibco), 1% AAS (Sigma-Aldrich)], ‘OLIGO’ [DMEM Glutamax high glucose (Gibco), 1% ITS (Gibco) 1% AAS (Sigma-Aldrich)], or ‘OLIGO + T3’ [DMEM Glutamax high glucose (Gibco), 1% ITS (Gibco) 1% AAS (Sigma-Aldrich) + T3 40 ng/ml (triiodothyronine, Sigma)].

For a complete list of reagents and materials used for cell culture, see Supplementary Table 1.

### RT-qPCR Gene Expression Analysis

To evaluate the expression of selected oligodendrocyte genes during cell culture in different media, reverse transcription-quantitative polymerase chain reaction (RT-qPCR) was used. The isolation of total RNA from cells on 6 DIV was performed using a column method, with the NucleoSpin RNA Plus XS kit (Macherey–Nagel) according to the manufacturer’s protocol. The kit included columns for genomic DNA removal; therefore, no DNA digestion procedure was required in subsequent sample preparation steps. The RNA obtained was suspended in nuclease-free water. The concentration and purity of RNA in the obtained samples were determined using a NanoDrop One spectrophotometer (ThermoFisher); samples whose value of the A260/A280 ratio exceeded 1.9, which indicated the purity of the obtained material, were used for further analyses. In the next step, a reverse transcription reaction was performed using 500 ng of RNA sample, modified MMLV reverse transcriptase (derived from Moloney murine leukemia virus reverse transcriptase) and primers in the form of a mixture of oligo(dT) and random hexamers (iScript cDNA Synthesis Kit from Bio-Rad). The reaction was carried out using an Eppendorf Mastercycler pro vapo.protect thermocycler according to the following protocol: priming (5 min/25 °C), reverse transcription (20 min/46 °C), and reverse transcriptase inactivation (1 min/95 °C). The cDNA was stored at − 20 °C. For the analysis of changes in gene expression using the qPCR technique, primer pairs for selected genes were designed using reference sequences from the National Center for Biotechnology Information (NCBI) database. The following genes were analysed: SRY-box transcription factor 10 (Sox10), oligodendrocyte transcription factor 2 (Olig2), 2′,3′-cyclic-nucleotide 3′ phosphodiesterase (Cnp) and myelin associated glycoprotein (Mag). The characteristics of the primers and their detailed sequences are listed in Supplementary Table 2. The primers were synthesized in the DNA Sequencing Laboratory of Institute of Biochemistry and Biophysics Polish Academy of Sciences. (Warsaw, Poland). The semi-quantitative PCR (qPCR) reaction was performed using an optimized iTaq Universal SYBR Green Supermix reaction mixture (Bio-Rad), containing iTaq hot-start DNA polymerase, deoxyribonucleotides, SYBR dye, and ROX reference dye. The reaction was performed using an Applied Biosystems 7500 Fast thermocycler according to the following protocol: polymerase activation and DNA denaturation (20 s/95 °C), 40 amplification cycles (denaturation 1 s/95 °C; annealing and plate read 20 s/60 °C), melting curve analysis (65–95 °C). Differences in gene expression were normalized with genes beta-2 microglobulin (B2m) and ribosomal protein L13; ribosomal protein L13 (Rpl13) and compared with measures obtained from samples collected from PROLIF medium, using the qBase model (Hellemans et al. 2007).

### Immunofluorescent Microscopy

To assess the phenotype of obtained oligodendrocyte lineage cells, cultures were fixed on days 1 for MGC cultures and days 3 and 6 for MB cultures. The culture medium was removed from the wells, these were then washed two times with PBS buffer. Then the cells were fixed in a solution of 4% paraformaldehyde in PBS at room temperature for 15 min.

The first step of immunofluorescent staining of fixed cells was to block the non-specific binding of primary antibodies. After 1 h incubation in the blocking mixture at room temperature, the coverslips were washed three times with a PBS solution and the primary antibody diluted in the blocking mixture was applied at different concentrations depending on the antibody used. After overnight incubation at 4 °C, the coverslips were washed three times with a PBS solution and the appropriate secondary antibodies diluted in PBS at 1:1000 were applied for 1 h of incubation at room temperature. When double staining was performed, the procedure was repeated using a second primary antibody from a different host. The composition of the blocking mixture and the antibodies used are summarised in Supplementary Table 3. Stained coverslips were washed three times with PBS solution and cell nuclei labelling solution, Hoechst 33258 (Sigma-Aldrich; 1:150 in PBS), was applied for 15 min. The coverslips were then washed again with PBS solution and mounted on a glass slide using a fluorescence mounting medium reagent (Dako). Microscopic images were acquired with the Cell Observer SD system (Zeiss).

### Statistical Analysis

To compare the efficiency of isolation the Test-*T* was used. Data for estimating the number of positively immunolabelled cells in microscopic images were obtained from three separate isolations and preparations, each with random 3–10 visual fields. Total cell counts were evaluated using the Hoechst cell nuclei marker. Statistical analysis of the qPCR results and quantification of oligodendrocyte lineage cells were performed using one-way analysis of variance (ANOVA) test with Tukey correction. Analyses were performed in GraphPad PRISM 8.0 software; differences at *p* < 0.05 were considered statistically significant.

## Results

The isolation efficiency was estimated by the number of cells obtained after shaking in the MGC method and after collecting the positive fraction in the MB method per head. The average numbers of cells were 924,545.00 ± 652,658.00 and 1,828,367.00 ± 1,001,069.00, respectively, and the difference was not statistically significant (test *T*, *p* = 0.0859).

**Fig. 2:**
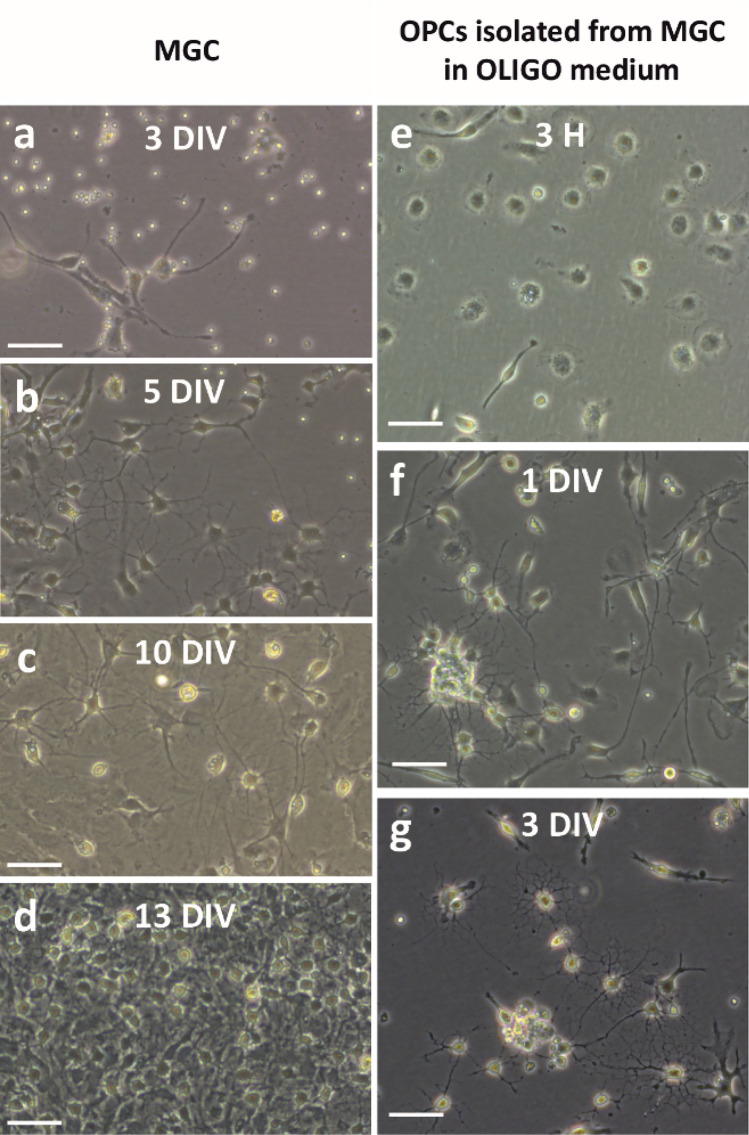
Live-cell images of cultures obtained with the MGC method. Mixed glial culture at 3 DIV (**a**), 5 DIV (**b**), 10 DIV (**c**), and 13 DIV (**d**) after the establishment of the culture. OPCs isolated from MGC, captured 3 h (**e**), 1 day, and 3 days (**g**) after seeding the cells. The scale bar corresponds to 50 µm

Cells of the mixed primary glial culture adhere to the bottom of the culture flask (Fig. 2a) and during the following days (Fig. 2b—5 days; Fig. 2c—10 days) proliferate intensely. They are ready for isolation of glial fractions after a dozen days (Fig. 2d—13 days). Cells of the oligodendrocyte lineage cultured in vitro have a characteristic morphology that it can be observed vividly under an inverted light microscope. This morphology changes as the cells differentiate and mature, therefore, even without performing immunofluorescence staining, the progress in culture maturation can be assessed. Freshly isolated cells attach to the bottom of the culture dish relatively quickly. Initially, cells have an amoeboid morphology, but after only a few hours they produce two protrusions, characteristic of cells at the progenitor stage. With the duration of the culture, the number of protrusions and their branching increases significantly (Fig. 2e–g). An increase in the complexity of the morphology of seeded cells, indicating progressive maturation from bipolar morphology, is observed already 3 h after seeding (Fig. 2e), through multiple branches 1 day after (Fig. 2f) to mature oligodendrocytes 3 days after (Fig. 2g).

**Fig. 3:**
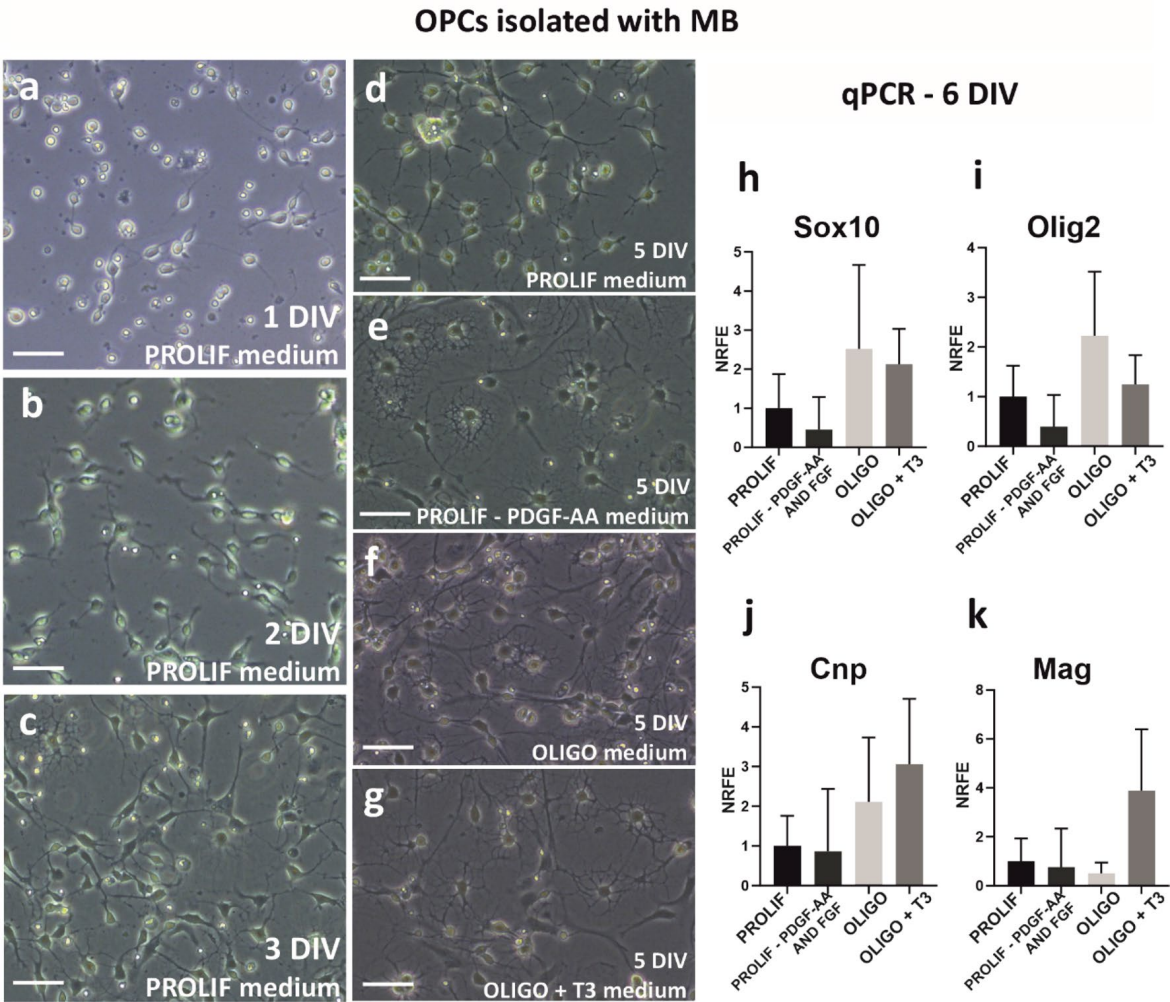
Live-cell images of cultures obtained with the MB method, cultured in PROLIF medium for 1 DIV (**a**), 2 DIV (**b**) and 3 DIV (**c**). **d**–**g** cells observed 5 DIV, cultured in different culture media for 2 days to induce oligodendrocyte differentiation: PROLIF medium (**d**), PROLIF – PDGFR-AA and FGF medium (**e**), OLIGO medium (**f**) and OLIGO + T3 medium (**g**). The scale bar corresponds to 50 µm. Changes in the expression of selected oligodendrocyte proteins, in cells cultured 6 DIV in different media, were evaluated by qPCR-measured mRNA level. Normalization was done against the results obtained in PROLIF medium (NRFE—normalized relative fold expression): Sox10 (**h**) and Olig2 (**i**) are expressed mainly in cells from the oligodendrocyte lineage at a progenitor stage. Cnp (**j**) is strongly expressed in immature oligodendrocytes, Mag (**k**) is a typical myelin protein expressed in mature oligodendrocytes. Results were analysed with two way ANOVA, but no significant changes were observed

Cells obtained with the MB method are plated directly after enzymatic digestion of tissue and magnetic separation, thus their morphology appears initially different at the beginning of their in vitro culturing. That is, round, opalescent cells predominate at day 1 after seeding (Fig. 3a) and it takes a longer time to attach to the culture dish. However, after 2 days of culture, numerous bipolar OPCs can be observed (Fig. 3b) and on day 3 in PROLIF medium these cells start to differentiate into mature oligodendrocytes (Fig. 3c).

The next step to optimize the method of OPCs isolation with magnetic microbeads was to test different culture media to maintain culture for the following days and induce differentiation. Cells cultured in PROLIF medium did not differentiate intensely but kept proliferate (Fig. 3d). The withdrawal of PDGF-AA and FGF from PROLIF medium turned out to be sufficient to stop proliferation and induce the production of multiple cell branches (Fig. 3e). Replacement of PROLIF with OLIGO medium after 3 days of culture triggers oligodendrocyte differentiation, but the ability to proliferation is still retained to some extent (Fig. 3f). The addition of T3 to OLIGO medium results in the greatest number of multi-branched oligodendrocytes (Fig. 3g). Despite the increase in complexity of cell morphology cultured in PROLIF-PDGF-AA and FGF medium, the expression of Sox10 (Fig. 3h) and Olig2 (Fig. 3i), which are the classical markers of OPCs and pre-oligodendrocytes, as well as Cnp (Fig. 3j) and Mag (Fig. 3k), which are expressed in differentiating and mature oligodendrocytes, did not increase at this time-point of in vitro culture. The highest expression of OPCs markers was observed in OLIGO medium (Fig. 3h, i), while the highest expression of myelin proteins was detected in OLIGO + T3 medium (Fig. 3j, k).

**Fig. 4:**
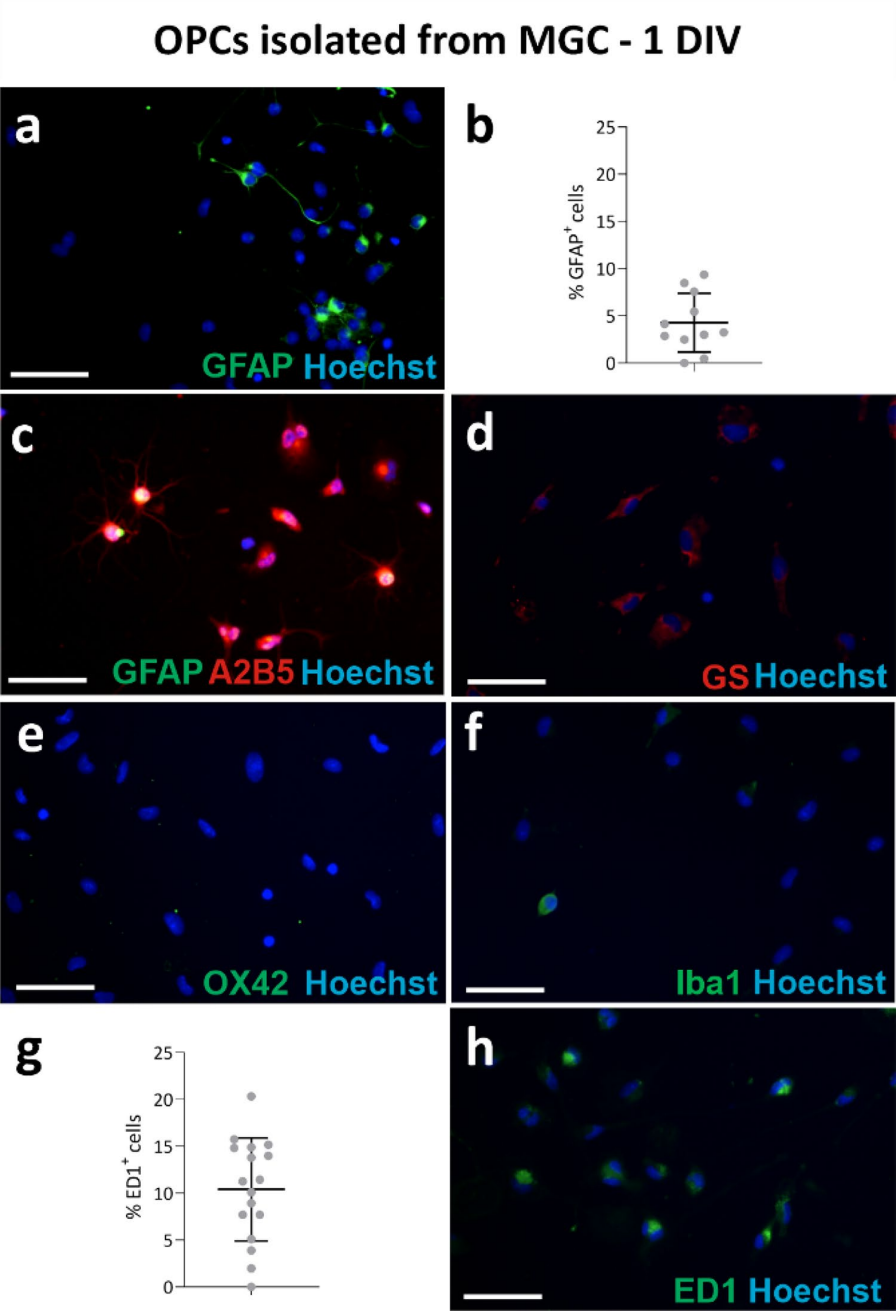
Immunofluorescent labelling of OPC cultures obtained with MGC method with astrocyte and microglia markers, 1 day after cell seeding; **a** GFAP+ cells identified as astrocytes; **b** results of the quantitative analysis of cells with GFAP expression and characteristic astrocytic cell morphology; **c** double staining with astrocyte marker anti-GFAP and OPC marker anti-A2B5; **d** staining with anti-glutamine synthetase (GS) marker; **e** no expression of the OX42 marker in cells in culture; **f** single Iba1 labelled cells in culture; **g** results of the quantitative analysis of cells with ED1 expression; **h** staining with anti-ED1 marker. Cell nuclei were labelled with Hoechst. The scale bar corresponds to 50 µm

**Fig. 5:**
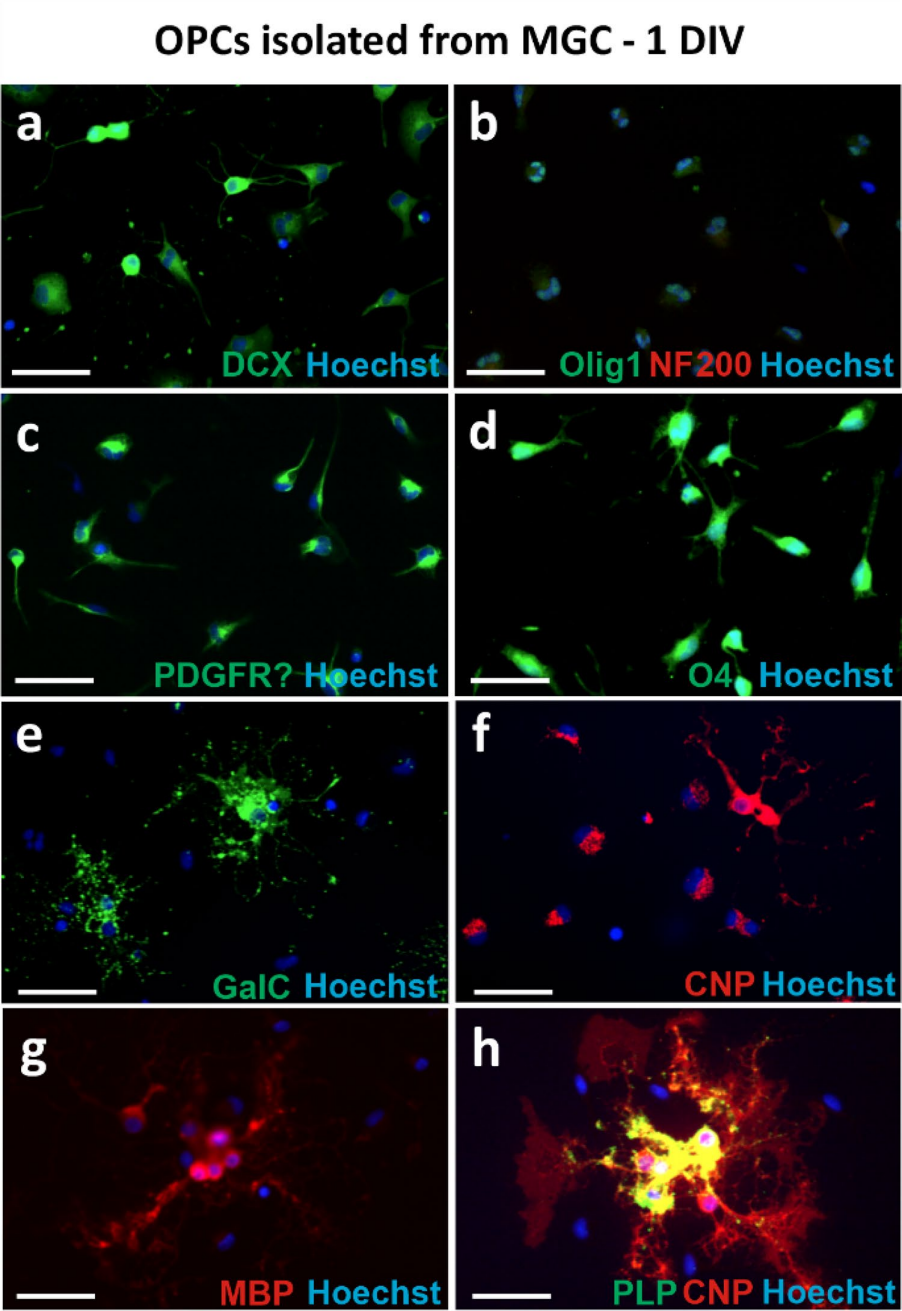
Immunofluorescent staining of OPCs cultures obtained with the MGC method with neuronal markers and oligodendrocyte lineage markers, fixed 1 day after cell seeding; **a** staining with an anti-DCX marker of immature neurons; **b** double staining with anti-NF 200 neuronal marker and anti-OLIG1 marker of pre-oligodendrocytes; **c** staining with anti-PDGFRα, marker of OPCs; **d** staining with the anti-O4 immature oligodendrocyte marker; **e** staining with anti-GalC immature oligodendrocyte marker; **f** staining with anti-CNP, immature oligodendrocyte marker; **g** staining with anti-MBP mature oligodendrocyte marker; **h** double staining of anti-CNP with a marker of mature oligodendrocyte anti-PLP. Cell nuclei were labelled with Hoechst. Scale bar corresponds to 50 µm

**Fig. 6:**
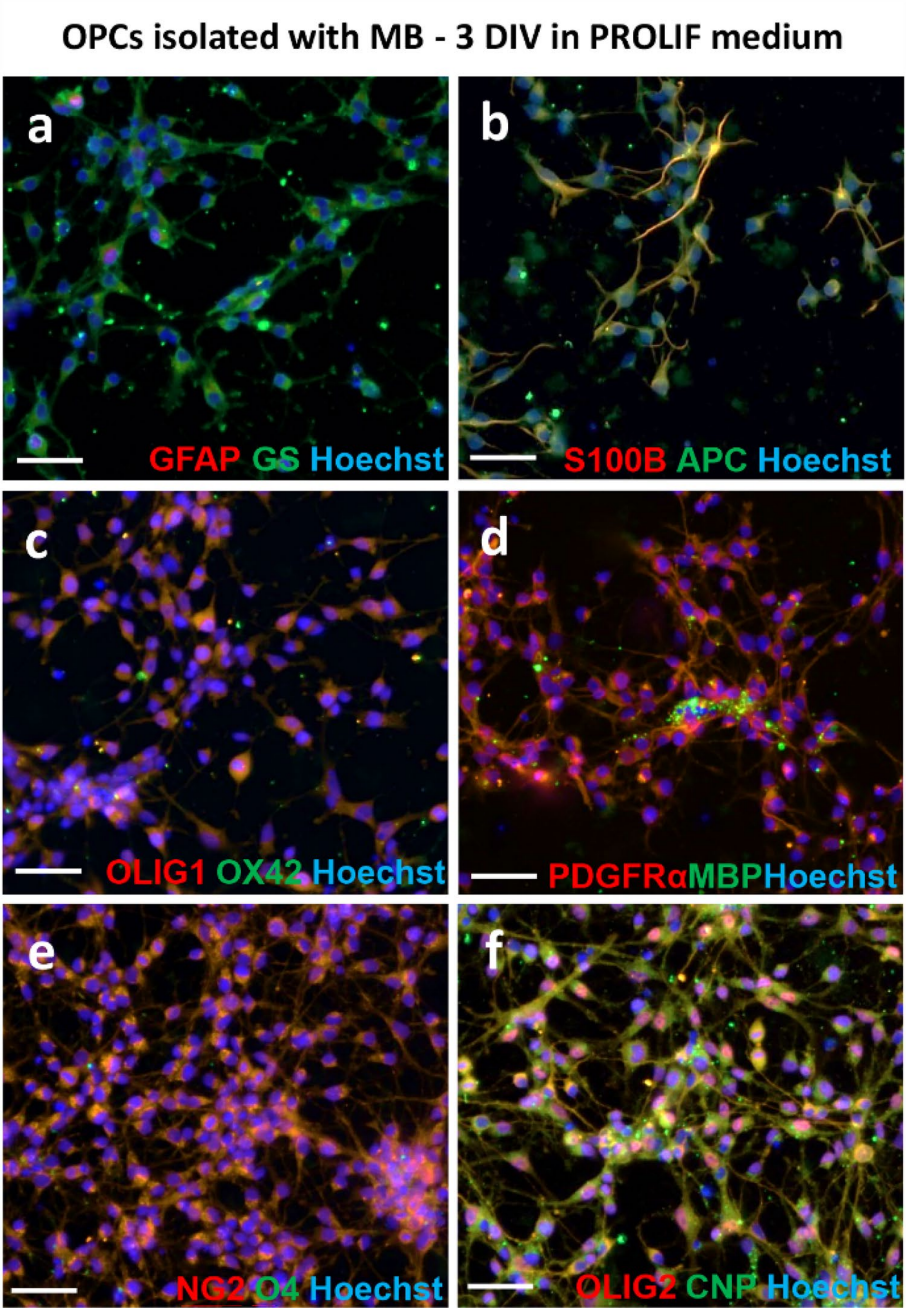
Immunofluorescent staining of cells obtained with MB method, fixed after 3 days of culturing in PROLIF medium; **a** double staining with GFAP and GS astrocyte markers; **b** double staining with S100β (astrocyte marker) and APC (oligodendrocyte marker); **c** double staining with OLIG1 (preoligodendrocyte marker) and OX 42 (microglia marker); **d** double staining with anti-PDGFRα (marker of OPCs) and MBP (mature oligodendrocyte marker); **e** double staining with NG2 (marker of OPCs) and O4 (marker of immature oligodendrocytes); **f** double staining with OLIG2 (preoligodendrocyte marker) and CNP (marker of immature oligodendrocytes. Cell nuclei were labelled with Hoechst. The scale bar corresponds to 50 µm

**Fig. 7:**
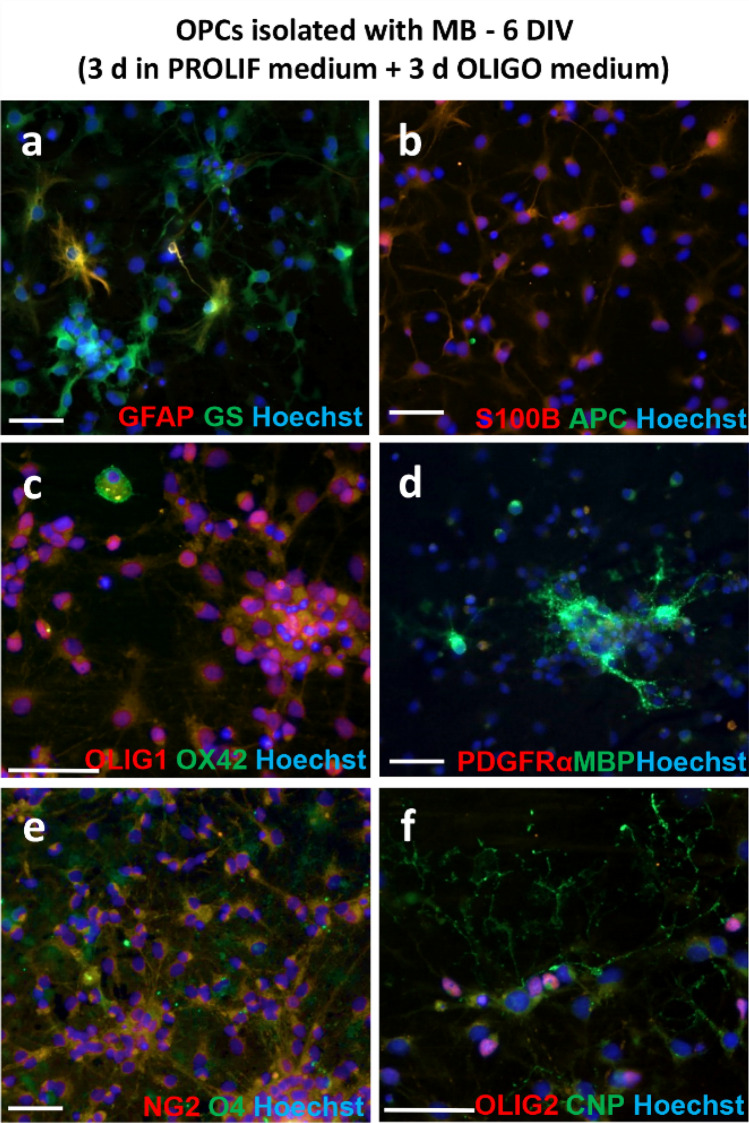
Immunofluorescent staining of cells obtained with MB method, fixed 6 DIV, after 3 days of culture in PROLIF and then 3 days in OLIGO medium; **a** double staining with GFAP and GS astrocyte markers; **b** double staining with S100β (astrocyte marker) and APC (oligodendrocyte marker); **c** double staining with OLIG1 (preoligodendrocyte marker) and OX 42 (microglia marker); **d** double staining with anti-PDGFRα (marker of OPCs) and MBP (mature oligodendrocyte marker); **e** double staining with NG2 (marker of OPCs) and O4 (marker of immature oligodendrocytes); **f** double staining with OLIG2 (preoligodendrocyte marker) and CNP (marker of immature oligodendrocytes. Cell nuclei were labelled with Hoechst. The scale bar corresponds to 50 µm

To evaluate the level of homogeneity of the OPCs isolated with different methods, immunofluorescence staining was performed on cultures fixed 1 day after seeding the cells with MGC method (Figs. 4, 5) or after 3 days in PROLIF medium (Fig. 6) and after next 3 days in OLIGO medium (Fig. 7) after seeding the cells obtained with MB method.

To assess the presence of contaminating astrocytes in culture, the slides were stained with a widely used marker for these cells, anti-GFAP, which recognises the glial fibrillary acidic protein (Cahoy et al. 2008). Progenitor cells have been shown to express this marker, both in vivo and in vitro (Itokazu et al. 2016; Salinas Tejedor et al. 2015). Therefore, an examination of the characteristic cell morphology was employed as an additional parameter evaluated. Consequently, GFAP+ cells with typical star-shaped morphology, with GFAP expression within long cytoplasmic processes (Fig. 4a), were identified as astrocytes. These cells represented 4.28% ± 0.93% of the culture isolated with the MGC method (Fig. 4b). Double staining with the anti-A2B5 glial progenitor marker demonstrated GFAP expression within the cell body of branched A2B5+ cells (Fig. 4c). Cultures obtained with the MB method did not express GFAP after 3 days of maintaining them in PROLIF medium (Fig. 6a), however, single GFAP^+^ cells were observed after culturing cells OLIGO medium (Fig. 7a). Other common markers of astrocytes used in this study were glutamine synthetase (GS) and S100β. The first is an enzyme detected mainly in astrocytes (Rocchio et al. 2019); however, its production by oligodendrocytes has also been reported (Bernstein et al. 2014; Xin et al. 2019). Calcium-binding protein S100β was also recently reported to be expressed in progenitor cells, as well as in the mature oligodendrocytes; therefore, the use of aforementioned proteins as selective astrocyte markers should be questioned (de los Angeles Castillo-Rodriguez et al. 2022; Du et al. 2021; Su et al. 2021). In the following experiments, low expression of GS was observed in the MGC-derived cultures (Fig. 4d). The OPCs obtained with the MB method expressed S100β and GS during culture in PROLIF medium (Fig. 6a, b), as well as later in OLIGO medium, but the labelled cells did not represent the classical astrocyte morphology (Fig. 7a, b).

To evaluate the content of microglial cells, staining was performed with standard markers of these cells, i.e., anti-OX42, anti-Iba1 and anti-ED1 (CD68). None of the obtained with MGC method cultures showed OX42+ cells labelling of the typical microglia and macrophage antigen, CD11b/c (Floden and Combs 2007) (Fig. 4e), as well as from the MB method (Fig. 6c). Single cells OX42+ were found in cells cultured in OLIGO medium (Fig. 7c). In MGC-derived cultures single cells expressing Iba1, an ionised calcium-binding adapter molecule 1, which is the activated microglia marker (Nakano et al. 2020) (Fig. 4f) were also observed. 10.4% ± 1.33% of the cells in culture were labelled with anti-ED1 (Fig. 4g, h). The anti-ED1 antibody recognizes a glycosylated protein characteristic of most macrophage populations (Dijkstra et al. 1985).

Staining with anti-DCX and anti-NF200 markers was used to evaluate the potential presence of neurons in cultures obtained with the MGC method. Almost all cells in the culture showed DCX expression to varying degrees (Fig. 5a). DCX, or doublecortin, is a microtubule-associated protein of neural progenitor cells used as a marker of immature neurons. Expression of this protein is also confirmed in oligodendrocyte progenitor cells, where it plays a role in cell migration (Boulanger and Messier 2017). However, no cells labelled with anti-NF200 antibody, a marker of intermediate filaments present in the cytoplasm of mature neurons, were observed in the double staining with anti-OLIG1 performed (Lin et al. 2014) (Fig. 5b).

To confirm the oligodendroglial phenotype of cells in cultures obtained with different methods, staining with classical oligodendrocyte markers at different stages of maturation was performed (Baumann and Pham-Dinh 2001). Most of the cells in the cultures obtained with the MGC method (Fig. 5c) as well as with the MB method cultured in PROLIF medium (Fig. 6d) showed expression of the α receptor for PDGF (PDGFRα), characteristic for oligodendrocyte progenitor cells. Its expression disappeared in MB cultures after culturing cells in OLIGO medium (Fig. 7d). Cells obtained with the MB method were also labelled with the OPC marker NG2 and its strong expression was observed in medium PROLIF (Fig. 6e). On the 1st day (MGC method) or 3 days after seeding (MB method), the majority of the cells expressed also oligodendrocyte transcription factors OLIG1 (Figs. 5b, 6c, 7c) and OLIG2 within nuclei (Figs. 6f, 7f) and the cells with the expression of O4, a marker of pre-oligodendrocytes predominated (Figs. 5d, 7e). The cells labelled with anti-GalC (galactocerebroside) and anti-CNP (2′,3′-cyclic-nucleotide 3′-phosphodiesterase), which are considered classical markers of immature oligodendrocytes were also observed (Fig. 5e, f). It was also observed in MB cultures that CNP can be expressed in cells cultured in PROLIF medium (Fig. 6e) even in cells without multiple processes, but directed toward oligodendrocyte maturation. This observation was later confirmed in cultures maintained in OLIGO medium, where CNP expression was present in branched oligodendrocytes (Fig. 7f). Mature oligodendrocytes with expression of the myelin basic protein (MBP) and the proteolipid protein (PLP) were also detected in cells from the MGC method on the 1st day of culture (Fig. 5g, h). MBP was not widely expressed at the beginning in cultures from the MB method (Fig. 6d), but it was observed more often after the following days of culture in OLIGO medium (Fig. 7d). Another marker, anti-adenomatosis polyposis coli protein (APC), which labels the cell body of myelinating oligodendrocytes, was also evaluated in cultures obtained with the MB method. It is widely used to detect oligodendrocytes in neural tissue, but not often reported in in vitro cultures (Lang et al. 2013). Interestingly, in the following study it was detected only within cells cultured in PROLIF medium (Fig. 6b), but not in the OLIGO medium (Fig. 7b).

**Fig. 8:**
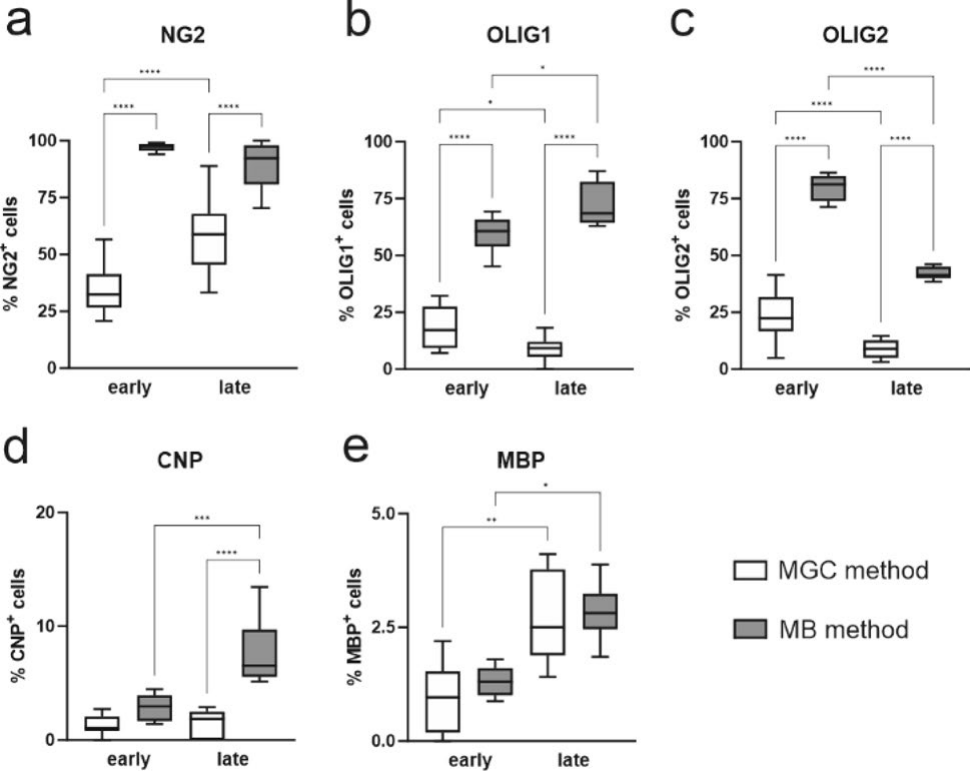
Quantification of immunolabelled oligodendrocyte lineage cells in cultures obtained with different methods at the early and late stages of their differentiation. Cells isolated with the MGC method were cultured in OLIGO medium and fixed at 2 DIV (early stage) and 5 DIV (late stage, respectively). Cells obtained with the MB method were fixed at 3 DIV (cultured in PROLIF medium; early stage) and 6 DIV (3 days in PROLIF and 3 days in OLIGO medium; late stage). OPCs were identified with anti-NG2 (**a**), pre-oligodendrocytes were labelled with anti-Olig1 (**b**) and anti-Olig2 (**c**), immature oligodendrocytes were identified with anti-CNP (**d**), and mature oligodendrocytes were identified with anti-MBP (**e**). The statistical significance of the obtained results was evaluated by a one-way analysis of variance (ANOVA) test with Tukey correction. Statistically significant differences *p < 0.05; **p < 0.01; ***p < 0.001 ****p < 0.0001

Detailed characterization of the oligodendrocyte culture, based on the expression of typical lineage markers on cells obtained with MGC method has been provided previously (Janowska et al. 2018). The analysis of the former results with the comparison to the quantification of cells obtained with MB method in the following study brings up some interesting observations. Using MB isolation, we can obtain a higher percentage of progenitor cells in culture, which is likely due to the elimination of contaminants from other glial fractions, present in cultures established with the MGC method. At early stage of differentiation there is almost three times more NG2+ OPCs in cultures form MB method than in cultures from MGC method of isolation (Fig. 8a, *p* < 0.0001). With MB method, the cultures enriched with pre-oligodendrocytes, expressing OLIG1 and OLIG2 within the nuclei are obtained (Fig. 8b, c). There is no difference between methods in the number of immature oligodendrocytes obtained at the early stage of culture growth (Fig. 8d), but at the late stage there is more than 500% increase in CNP expression in cultures obtained with MB method compared to MGC (Fig. 8d, *p* < 0.0001). Nonetheless, regardless of the method used, we observe a progression in differentiation in the form of increasing the proportion of cells with the expression of MBP, corresponding to mature oligodendrocytes (Fig. 8e; MGC *p* < 0.01; MB *p* < 0.05).

## Discussion

The basis for the isolation and culture of rat oligodendrocyte progenitor cells employing the MGC method was developed in the 1980s by McCarthy and de Vellis (1980). The researchers observed that in cultures obtained from minced rat brain cortex, a specific stratification of different cell types occurs over several days of culture, where astrocytes populate the bottom of the culture dish, while oligodendrocyte progenitor cells, oligodendrocytes and microglia grow on their layer. To further separate each fraction, a method of shaking the culture dishes has been proposed, which takes advantage of the different adhesive properties of OPCs and microglia. Microglia are most easily isolated from such a culture, after only about 1 h of orbital shaking at 200 rpm (Tamashiro et al. 2012), or even after tapping the culture bottle against the laminar chamber top (Lian et al. 2016). After this, the culture medium is replaced with fresh one, and the collected supernatant is centrifuged to obtain microglia cells for experiments or discarded. To further isolate OPCs from the mixed culture, the culture bottles are shaken again for several hours at the same speed and the obtained supernatant is subjected to an additional step of OPCs purification. The cells that remain in the culture dish after all shaking steps are astrocytes (Chen et al. 2007; Wang et al. 2012). The speed and shaking time of the mixed cultures can determine the purity of the resulting OPCs. The most commonly used protocol, described in detail by Chen et al. in 2007, applies a speed of 200 rpm for 18–20 h. However, the high speed forces the use of a tight seal on the culture bottles to prevent leakage of the culture medium. Therefore, cultures are maintained for all this time without regulation of CO_2_ concentration, which contributes to a rapid increase in the pH of the medium and can adversely affect cell viability and biology. In this study, the reduced shaking speed (160 rpm) and culture flasks with a filter cap were used, which allowed the cultures to be maintained in a CO_2_-regulated atmosphere throughout the procedure. In the standard McCarthy and de Vellis method, an additional step is used to “purify” the obtained supernatant containing OPCs by incubating it for about an hour in a non-coated Petri dish (McCarthy and de Vellis 1980). The assumption is that astrocytes and microglia are the cells that will stick more easily to the bottom of the dish, while OPCs characterized by low adhesion, will remain suspended. However, in subsequent attempts to apply this method, no significant differences in adhesion abilities were observed at this stage of cell isolation. The additional procedure further increased the time and cost of cell isolation, and finally this step was omitted. Nevertheless, the use of a lower shaking rate and the maintenance of cultures with adjustable concentrations of oxygen and carbon dioxide during shaking, yielded in cultures of satisfactory purity, in which more than 86% of the cells were identified as oligodendrocytes.

Isolation methods that provide high purity cell fractions with high homogeneity include techniques based on isolating cells from the homogenate of brain hemispheres using labelling with cell-specific antibody. To perform a positive selection of OPCs one would need to use antibodies directed against membrane antigens of these cells such as A2B5, PDGFRα, or NG2, and in the case of selection of immature oligodendrocytes, the O4 antibody. Immunopanning is one of the labelling-based techniques. The method involves incubating a suspension of cells in a Petri dish coated with a suitable antibody to select the desired fraction. Immunopannig, however, has lower efficiency and lower viability compared to the traditional method (Barres 2014; Emery and Dugas 2013). A modification of this method is to label cells directly with antibodies, followed by cell sorting. Cells labelled with fluorochrome-conjugated antibodies can be isolated using a flow cytometer (FACS—Fluorescence-Activated Cell Sorting). In this case, however, the potential phototoxic effect, which may affect the quality of the obtained fractions, must be taken into account. The particular sensitivity of OPCs to light emitted by LED lamps was recently described by Stockley et al. (2017). An alternative method is the sorting of cells labelled with magnetic beads, which is performed on columns placed in a magnetic field (MACS—Magnetic-Activated Cell Sorting) (Cizkova et al. 2009; Dincman et al. 2012). The material used to produce such beads is inert to cells and biodegradable, allowing them to be used in cell isolation for in vitro culture, according to manufacturer’s specification. A serious limitation of the latter two methods is the necessity to perform the isolation procedure under sterile conditions, which can be difficult, especially with a large FACS apparatus. Thus, the method developed in this study, which is based on the enzymatic digestion of brain tissue, as well as the magnetic cell sorting, performed under the hood, provides appropriate conditions for the subsequent use of obtained cells for in vitro culture. The limitation of the method is the ability of A2B5^+^ selected precursors to differentiate into type 2 astrocytes, especially in serum-rich medium (Fok-Seang and Miller 1992). It was previously shown that use of serum-free medium reduces the growth of astrocytes and fibroblasts in OPCs culture in vitro (Bottenstein 1986; Emery and Dugas 2013; Sakurai et al. 1998).

To promote cell proliferation and oligodendrocyte lineage phenotype, in the following study, isolated cells were cultured directly in serum-free medium supplemented with PDGF-AA and bFGF for the first 3 days after seeding. After treatment with the aforementioned mitogens, OPCs are characterized by a small size (6–12 µm in the case of neonatal rat cells), a clearly visible, rounded cell body, the presence of few processes and a high proliferation capacity (Wu et al. 2011). We showed that supplementation of culture medium with PDGF-AA and bFGF significantly increases the proportion of progenitor cells and cells at the initial stages of differentiation in culture. PDGF is considered a major proliferation factor for OPCs, involved in the activation of cyclin-dependent kinase 2 (Cdk2), a cell cycle regulator (Boulanger and Messier 2014; Paez et al. 2006). The molecule that enhances the effect of PDGF is bFGF. This factor causes an increase in the expression of receptors for PDGF (i.e. PDGFαR) on the cell membrane surface, and additionally acts as an inhibitor of cell differentiation (McKinnon et al. 1990). Deprivation of the medium of the aforementioned growth factors promoted the initiation of cell differentiation. In addition, to increase the efficiency of the cell maturation process, the medium is often supplemented with the thyroid hormone T3 at a concentration of 15–30 nM (Baas et al. 1997; Schoor et al. 2019). T3 interacts with nuclear receptors, including TRβ, and thus regulates the expression of genes related to oligodendrocyte maturation and myelination (Baldassarro et al. 2019). Moreover, a common addition to the culture medium is fetal calf serum, which is a non-specific source of proteins, including growth factors, of undefined composition and concentrations, which may vary between batches of product. Therefore, it may not be possible to standardise cell culture conditions using serum. Culture medium supplements that allow serum reduction or elimination are becoming increasingly popular. One such supplement is ITS, whose main components are insulin (172 nM), transferrin (6.8 nM) and sodium selenite (3.8 nM). The use of a supplement based on the aforementioned components in oligodendrocyte culture was documented as early as 1986 thanks to the work of Bottenstein (1986). The researcher showed that, compared to medium enriched with 5% FBS, supplementation with ITS components increased the maturation of oligodendrocytes.

**Fig. 9:**
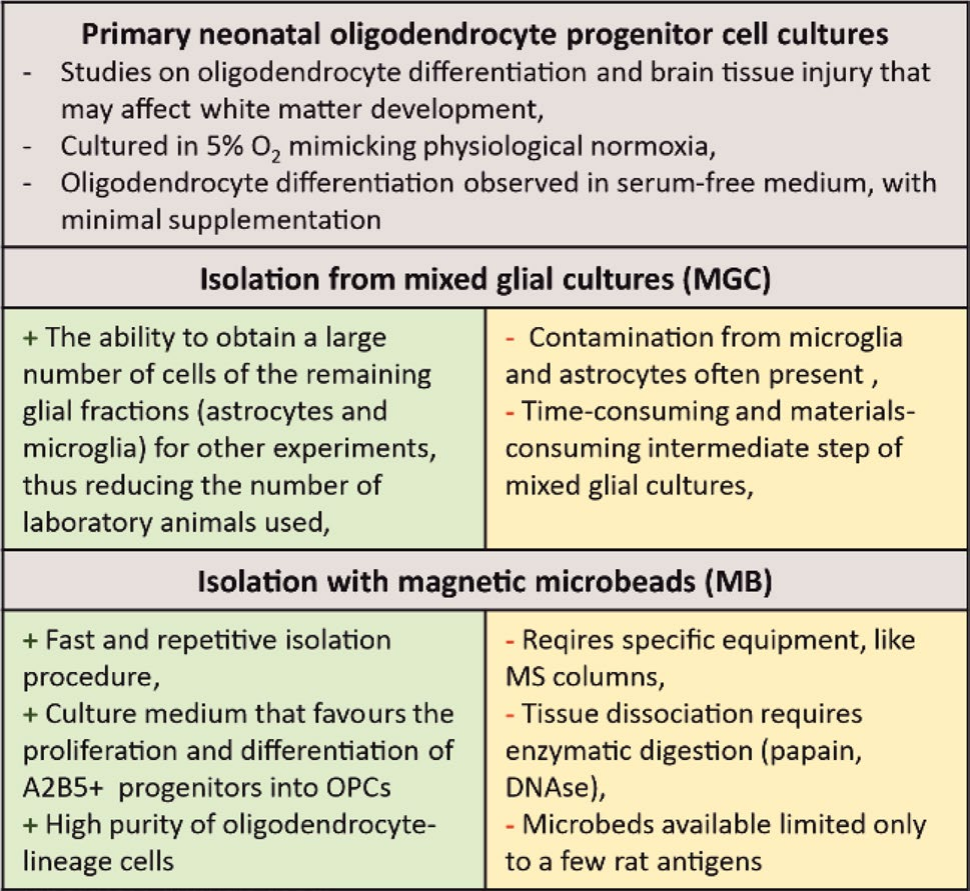
A summary of the most important features of rat primary OPCs cultures, advantages (+) and disadvantages (−) of the isolation methods used in the study

In our previous study, we showed that keeping cultures in normoxic conditions (5% O_2_) allowed to have cultures with greater number of OPCs and higher rate of proliferation than cells cultured in atmospheric oxygen level (Janowska et al. 2018). Here we showed, that on the 1st day of culture obtained with MGC and on the 3rd day of culture established with the MB method, the majority of cells expressed the oligodendrocyte progenitor marker PDGFRα and/or NG2, as well as OLIG1 and OLIG2 markers. Lower cell count expressed the immature oligodendrocyte markers O4 or GalC. Mature oligodendrocytes with an expression of the myelin proteins MBP and PLP were already observed on the 1st day of a culture of cells from the MGC method. Single cells expressing MBP were also observed in cells isolated with MB method on 3 DIV in PROLIF medium. The literature review shows that the optimized MGC method applied in this study allows obtaining cells with similar phenotype as the cells obtained by the traditional protocol. Authors usually report that the proportion of cells with expression of markers typical for OPCs obtained in such cultures is 50–95% (Mazuir et al. 2020; O’Meara et al. 2011; Schott et al. 2016; Yang et al. 2005). Some do not present the purity and characteristics of the culture in which they perform subsequent experiments, and this may be important for the correct interpretation of the results. When choosing a method to isolate OPCs for testing, it is worth considering the advantages and disadvantages of both methods (Fig. 9). Although the yield of cells per animal is similar, the isolation time is significantly reduced, from more than 10 days for the MGC method to a few hours for the MB method. Hence, the consumption of FBS-containing culture medium to maintain a mixed glial culture and the cost of maintaining the incubator are reduced. On the other hand, there is the cost of purchasing magnetic beads and columns, as well as a kit for enzymatic dissociation of the tissue in the MB method. More importantly, the cultures obtained by the MGC method have contaminants in the form of macrophages/microglia and a high number of astrocytes, which are in turn rarely observed in cultures obtained by the MB method.

To summarize, the detailed protocols presented in this study can be used to obtain oligodendrocyte progenitor cells from neonatal rats in order to model oligodendrogenesis and oligodendrocyte maturation in vitro. The choice of the optimal isolation method depends on further research plans and the availability of equipment. It should be noted that the use of reduced oxygen concentration in the culture incubator used, which better mimics physiological conditions, allowed us to obtain viable cultures that differentiate properly within a couple of days of culture in serum-free medium. Not using the addition of serum in the culture medium will allow various pharmacological compounds to be tested in these models, especially those designed to treat brain disorders in neonatal period, and reproducible results between different research groups.

## Supplementary Information

Below is the link to the electronic supplementary material.

Supplementary file1 (DOCX 37 kb)

## Data Availability

Enquiries about data availability should be directed to the authors.
