# Supplementary material for: Pearls and Pitfalls of Isolating Rat OPCs for In Vitro Culture with Different Methods
Source: Cell Mol Neurobiol. 2023 Jul 5;43(7):3705–22. doi: 10.1007/s10571-023-01380-2 (PMC10477124; doi:10.1007/s10571-023-01380-2)
Supplement: Supplementary file 1 — Supplementary file1 (DOCX 37 kb) [file 10571_2023_1380_MOESM1_ESM.docx]

Supplementary material

Pearls and pitfalls of isolating rat OPCs for *in vitro* culture with different methods. *Cellular and Molecular Neurobiology*; Justyna Janowska^1^, [jjanowska@imdik.pan.pl](mailto:jjanowska@imdik.pan.pl); Justyna Gargas^1^, [jgargas@imdik.pan.pl](mailto:jgargas@imdik.pan.pl) Joanna Sypecka^1^, [jsypecka@imdik.pan.pl](mailto:jsypecka@imdik.pan.pl) (corresponding author)

**Table 1. List of reagents and materials used for cell culture.**

| Name | Manufacturer | Catalog number |
| --- | --- | --- |
| AAS – antibiotic-antimycotic solution | Sigma | A5955 |
| anti-A2B5 magnetic microbeads | Miltenyi | 130-093-388 |
| BSA - bovine serum albumin | Sigma | A7030 |
| cell strainer 40 µm | pluriSelect | 43-57040-03 |
| cell strainer 70 µm | Miltenyi | 130-098-462 |
| connector ring for cell strainer 40 µm | pluriSelect | 41-50000-03 |
| culture flask Nunc EasY T75 | ThermoFisher | 156499 |
| culture plate Nunc 24-well | ThermoFisher | 142475 |
| DMEM Glutamax high glucose | Gibco | 10566016 |
| FBS – fetal bovine serum | Gibco | 10500064 |
| FGF-2 – fibroblast growth factor -2 (mouse). | Miltenyi | 130-105-787 |
| HBSS (w) (Hank’s balanced salt solution with calcium and magnesium | Gibco | 14025092 |
| HBSS (w/o) (Hank’s Balanced Salt Solution without calcium and magnesium | Gibco | 14175095 |
| ITS – insulin-transferrin-selenium | Gibco | 41400045 |
| L-glutamine | Sigma | G7513 |
| MS columns | Miltenyi | 130-042-201 |
| MACS Neuro Medium | Miltenyi | 130-093-570 |
| Neural Tissue Dissociation Kit P | Miltenyi | 130-092-628 |
| OctoMACS Separator | Miltenyi | 130-042-109 |
| PDGFAA - platelet-derived growth factor alpha polypeptide a (human) | Miltenyi | 130-093-977 |
| PBS - phosphate buffered saline | Gibco | 18912-014 |
| poly-L-lysine (hydrobromide) | Sigma-Aldrich | P6282 |
| T3 - triiodothyronine | Sigma | T2877 |

**Table 2. List of primers used for qPCR.**

| Tested gene | Primer sequences | |
| --- | --- | --- |
| Cnp | F: 5’ GACCTGGTCAGCTATTTTGGC 3’ | R: 5’ GGCCTTGCCGTAAGATCTCC 3’ |
| Mag | F: 5’ TCCTGATTGCCATTGTCTGCT 3’ | R: 5’ AGAGATTCGGAATTCGGGGC 3’ |
| Olig2 | F: 5’ AGCGAGCACCTCAAATCGAA 3’ | R: 5’ AAGATCATCGGGTTCTGGCG 3’ |
| Sox10 | F: 5’ GGCTCACTACAAGAGTGCCC 3’ | R: 5’ CTGTCTTTGGGGTGGTTGGA 3’ |
| B2m | F: 5’ CGGGGTGGTGATGAGAAGTT 3’ | R: 5’ AAGGCTCCTTGTCCCTTGAC 3’ |
| Rpl13 | F: 5’ GAAGAAGGGAGACAGTTCTGC 3’ | R: 5’ AGTTCTTCTCCTCTTCCGTGATG 3’ |

**Table 2.** Primer sequences designed with Primer-BLAST software was used to design primer pairs characterized by the following properties: primer pairs separated by at least 1 intron in the corresponding genomic DNA (except Olig2); length of primers 19-23 bases; PCR product length 75-150 bases; melting temperature of the primers 59-60^o^C; proportion of GC bases 55-60% of the sequence. The specificity of the designed primer pairs was verified in the Nucleotide BLAST software.

**Table 3. List of reagents used for immunofluorescent microscopy.**

| Blocking solution | | | PBS (Gibco), normal goat serum (Sigma) 10%, Triton X-100 (Serva) 0,1% | |
| --- | --- | --- | --- | --- |
| Primary antibodies | | | | |
|  |  | ***Target antigen*** | | ***Antibody*** |
|  | **Oligodendrocyte progenitor cells (OPCs)** | **NG2** - chondroitin sulfate proteoglycan 4 | | Rabbit IgG anti-NG2 (1:200)  (SantaCruz; sc-20162) |
|  |  | **A2B5** - cell surface ganglioside epitope | | *Mouse IgM anti-A2B5 (1:200)  (Chemicon; MAB312R) |
|  |  | **PDGFRα** – type α receptor for platelet-derived growth factor | | Rabbit IgG anti‑PDGFRα  (1:500) (SantaCruz; sc-338) |
|  | **Pre- oligodendrocytes**  **(pre-OLs)** | **OLIG1** - basic-helix-loop-helix transcription factor | | Rabbit IgG anti-Olig1 (1:1000)  (MERCK; AB15620) |
|  |  | **OLIG2** - basic-helix-loop-helix transcription factor | | Rabbit IgG anti-Olig2 (1:500)  (MERCK; ABN899) |
|  | **Immature oligodendrocytes (i‑OLs)** | **CNPase** - 2',3'-cyclic-nucleotide 3'-phosphodiesterase | | Mouse IgG1 anti-CNPase (1:100)  (Chemicon; MAB326) |
|  |  | **GalC** – galactocerebroside | | *Mouse IgG3 anti-GalC (1:200)  (Chemicon; MAB342) |
|  |  | **O4** – O-antigen, a sulfated galactocerebroside | | Mouse IgM anti-O4 (1:100)  (Merck; MAB345) |
|  | **Myelinating oligodendrocytyes (OLs)** | **MBP** – myelin basic protein | | Mouse IgG1 anti-MBP (1:100)  (Chemicon; MAB381) |
|  |  | **PLP** – proteolipid protein | | Mouse IgG2a anti-PLP (1:100)  (Chemicon; MAB388) |
|  |  | **APC** - adenomatosis polyposis coli protein | | Mouse IgG2b anti-APC (1:100) (abcam; ab16794) |
|  | **Microglia** | **Iba-1** - ionized calcium-binding adapter molecule 1 | | Goat IgG anti-Iba1 (1:400)  (abcam; ab107159) |
|  |  | **ED1** – CD68 protein of a specific clone | | Mouse IgG1 anti-ED1 (1:400)  (BioRad; MCA341R) |
|  |  | **OX42** – antibody against membrane CD11 b and CD11c | | Mouse IgG2a anti-OX42 (1:200)  (abcam; ab1211) |
|  | **Astrocytes** | **GFAP** – glial fibrillary acidic protein | | Rabbit IgG anti-GFAP (1:200)  (Dako; 20334) |
|  |  | **GS** - glutamine synthetase | | Mouse IgG1 anti‑GS (1:200) (abcam; ab64613) |
|  |  | **S-100β** - calcium-binding protein β | | Rabbit IgG S-100β (1:200)  (Dako; ab52642) |
|  | **Neurons** | **NF200** – neurofilament 200 | | Mouse IgG1 anti-NF200 (1:400)  (Sigma; N0142) |
|  |  | **DCX** - doublecortin | | Goat IgG anti-DCX (1:200)  (SantaCruz; SC-8066) |
| Secondary antibodies | | | | |
|  | | Goat anti-rabbit IgG (H+L) Alexa Fluor 488 (Invitrogen; A-11008) | | |
|  |  | Goat anti-rabbit IgG (H+L) Alexa Fluor 546 (Invitrogen; A-11035) | | |
|  |  | Donkey anti-goat IgG (H+L) Alexa Fluor 488 (Invitrogen; A-11055) | | |
|  |  | Goat anti-mouse IgG1 Alexa Fluor 488 (Invitrogen; A-21121) | | |
|  |  | Goat anti-mouse IgG1 Alexa Fluor 546 (Invitrogen; A-21123) | | |
|  |  | Goat anti-mouse IgG2a Alexa Fluor 488 (Invitrogen; A-21131) | | |
|  |  | Goat anti-mouse IgG3 Alexa Fluor 488 (Invitrogen; A-21151) | | |
|  |  | Goat anti-mouse IgM Alexa Fluor 546 (Invitrogen; A-21045) | | |

**Table 3.** Antibodies were used in single or double staining of fixed cell cultures, using the appropriate secondary antibody. *Triton X-100 was not added to the blocking mixture for in vitro cell staining according to the manufacturer's instructions.
